# Supplementary material for: The interactome of intact mitochondria by cross-linking mass spectrometry provides evidence for coexisting respiratory supercomplexes
Source: Mol Cell Proteomics. 2017 Dec 8;17(2):216–32. doi: 10.1074/mcp.RA117.000470 (PMC5795388; doi:10.1074/mcp.RA117.000470)
Supplement: Supplemental Data [file supp_RA117.000470_133922_0_supp_23978_fzffwf.zip › spectra_annotation/mito_spectra_annotation/439-1-4-1-32-1.pdf]

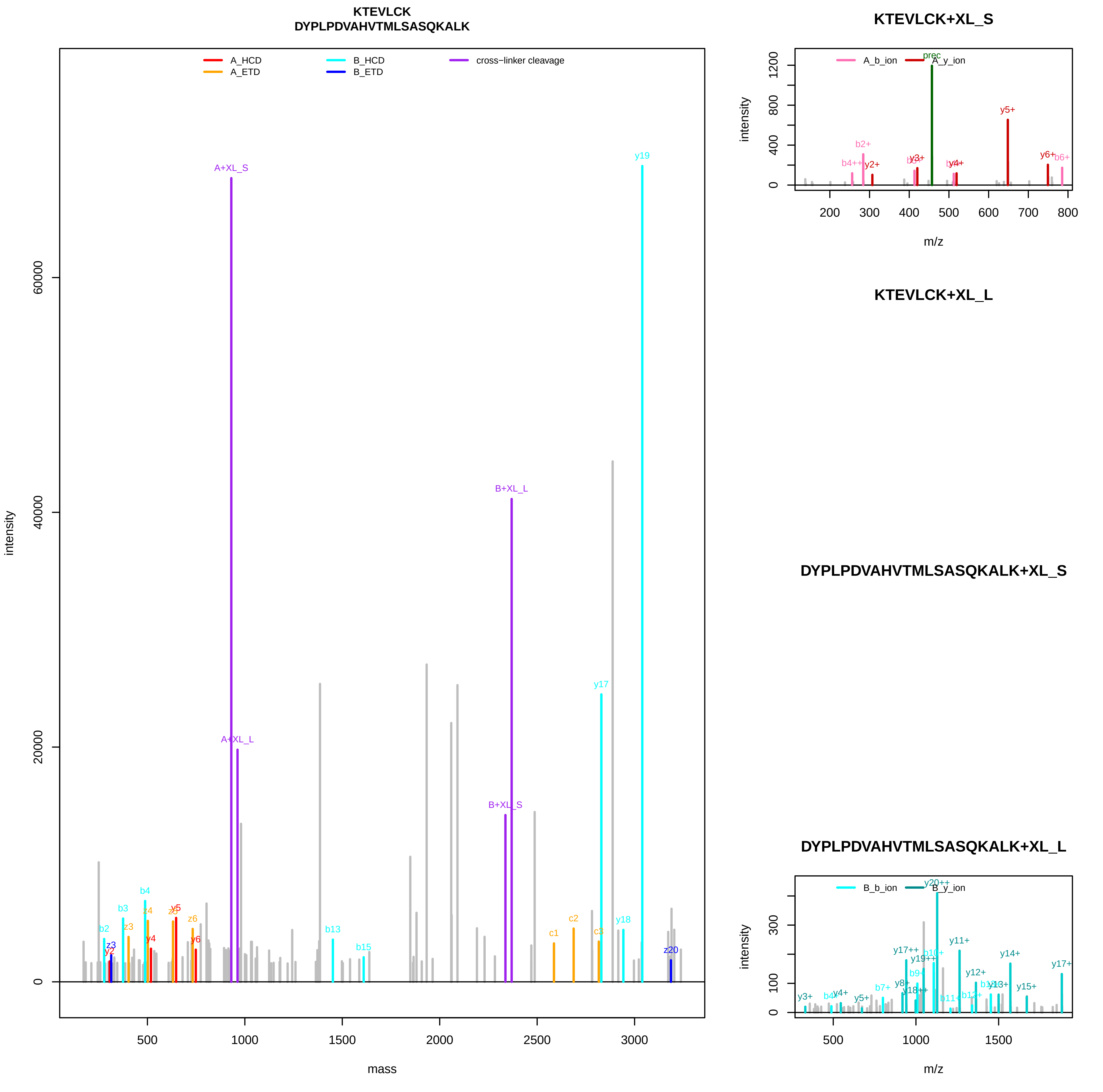
KTEVLCK
DYPLPDVAHVTMLSASQKALK
A_HCD
A_ETD
B_HCD
B_ETD
cross−linker cleavage
A+XL_S
A+XL_L
B+XL_L
B+XL_S
y19
y17
y18
b2
z3
y2
b3
z3
b4
z4
z5
y5
y4
z6
y6
b13
b15
c1
c2
c3
z20
intensity
mass
0
20000
40000
60000
500
1000
1500
2000
2500
3000
KTEVLCK+XL_S
A_b_ion
A_y_ion
prec
y5+
b2+
b4++
y2+
y3+
y4+
y6+
b6+
0
400
800
1200
200
300
400
500
600
700
800
m/z
KTEVLCK+XL_L
DYPLPDVAHVTMLSASQKALK+XL_S
DYPLPDVAHVTMLSASQKALK+XL_L
B_b_ion
B_y_ion
y20++
y11+
y17++
b10+
y19++
y14+
y17+
b9+
y12+
b13+
y8+
y18++
y15+
b7+
y3+
b4+
y4+
y5+
b11+
b12+
0
100
300
500
1000
1500
